# Determination of tissue-specific interaction between vitamin C and vitamin E *in vivo* using senescence marker protein-30 knockout mice as a vitamin C synthesis deficiency model

**DOI:** 10.1017/S0007114521004384

**Published:** 2022-09-28

**Authors:** Ayami Sato, Yuka Takino, Tomohiro Yano, Koji Fukui, Akihito Ishigami

**Affiliations:** 1 Molecular Regulation of Aging, Tokyo Metropolitan Institute of Gerontology, Tokyo 173-0015, Japan; 2 Institute of Life Innovation Studies, Toyo University, Gunma 374-0193, Japan; 3 Molecular Cell Biology Laboratory, College of Systems Engineering and Science, Graduate School of Engineering and Science, Shibaura Institute of Technology, Saitama 337-8570, Japan; 4 Department of Biological Sciences, Tokyo Metropolitan University, Tokyo 192-0397, Japan

**Keywords:** Ascorbic acid, *α*-tocopherol, Liver, Mice, SMP30, Vitamin C, Vitamin E

## Abstract

Vitamin E (*α*-tocopherol; VE) is known to be regenerated from VE radicals by vitamin C (L-ascorbic acid; VC) *in vitro*. However, their *in vivo* interaction in various tissues is still unclear. Therefore, we alternatively examined the *in vivo* interaction of VC and VE by measurement of their concentrations in various tissues of senescence marker protein-30 (SMP30) knockout (KO) mice as a VC synthesis deficiency model. Male SMP30-KO mice were divided into four groups (VC+/VE+, VC+/VE–, VC–/VE+ and VC–/VE–), fed diets with or without 500 mg/kg VE and given water with or without 1·5 g/l VC *ad libitum*. Then, VC and VE concentrations in the plasma and various tissues were determined. Further, gene expression levels of transporters associated with VC and VE, such as *α*-tocopherol transfer protein (*α*-TTP) and sodium-dependent vitamin C transporters (SVCTs), were examined. These results showed that the VE levels in the VC-depleted (VC–/VE+) group were significantly lower than those in the VC+/VE+ group in the liver and heart; the VC levels in the VE-depleted (VC+/VE–) group were significantly lower than those in the VC+/VE+ group in the kidneys. The *α*-TTP gene expression in the liver and kidneys was decreased by VC and/or VE depletion. Moreover, SVCT1 gene expression in the liver was decreased by both VC and VE depletion. In conclusion, these results indicate that VC spares VE mainly in the liver and heart and that VE spares VC in the kidneys of SMP30-KO mice. Thus, interaction between VC and VE is likely to be tissue specific.

Vitamin E (*α*-tocopherol; VE) is a lipid-soluble antioxidant acting as a peroxyl radical scavenging molecule that inhibits lipid peroxidation in biological membranes. *In vivo*, the OH group on the chromanol of VE is no longer available after reacting with a free radical and needs to be regenerated to maintain its antioxidant status^(1)^. VE can intercept lipid peroxyl radicals at the membrane surface as well as inside the membrane, and water-soluble agents that regenerate VE can access the chromanol group when it is located at the surface^(2)^.

In animals, dietary VE is absorbed from the small intestine along with other lipophilic nutrients and is integrated into chylomicrons^(3)^. Chylomicrons containing VE eventually reach the liver and are incorporated into hepatocytes by endocytosis. Then, the VE component is selectively secreted from the liver by *α*-tocopherol transfer protein. In hepatocytes, *α*-tocopherol transfer protein selectively binds VE and moves to the cell membrane^(4)^. VE is then transported to plasma lipoproteins by ATP-binding cassettes, subfamily A, member 1 (ABCA1) and is then distributed to peripheral tissues^(5,6)^.

Vitamin C (L-ascorbic acid; VC) is a water-soluble antioxidant discovered as an anti-scurvy factor^(7)^. Since reports of the possibility of VE regeneration from VE radicals by VC before 1980^(8)^, analyses of their interaction mechanism at the electron level have been conducted *in vitro*^(9)^. Experiments using cultured cells have provided evidence that VC spares VE^(10–12)^. Further, sparing of VE by VC in human LDL^(13)^, erythrocytes^(14)^ and platelets^(15)^ has also been indicated *in vitro* studies. Similar observations have been made in fish^(16)^. Several studies^(17–19)^ have also measured VC and VE concentrations in tissues after feeding depleted diets in inherently scorbutic (osteogenic disorder Shionogi) rats or guinea pigs. These studies suggested that VC interacts with VE in almost all tissues tested such as the liver, heart, lungs, kidneys and plasma. However, a lack of clarity remains regarding tissues that have not been examined or are inconsistent with the previously described results^(20,21)^. An examination of the effects of VC and VE supplementation on their respective plasma concentrations in human subjects showed results supporting these interaction^(22)^. Further, VC and VE supplementation for 3 to 6 years in hypercholesterolemic individuals is reported to slow atherosclerotic progression, but the interactive concentrations of VC and VE in the plasma are unclear^(23)^. Additionally, glutathione (GSH) level has not been considered in these previous *in vivo* studies that showed the interaction of VC and VE, although the GSH is known to regenerate VC^(24)^. Thus, the interaction of VC and VE *in vivo* is not simple and requires renewed evidence. However, the number of studies on the combination of these two vitamins has largely decreased in the last decade.

Previously, using senescence marker protein-30 (SMP30)/*α*TTP double-knockout (KO) mice, we revealed that VC and VE double-deficiency increases neuroinflammation and impairs conditioned fear memory^(25)^. These findings emphasise the importance of both VC and VE in biological functions. SMP30 was first discovered as an age-associated protein that decreases with age in the liver of rats and mice^(26,27)^. Subsequently, SMP30 was identified as a gluconolactonase, catalysing the reaction of L-gulonic acid to L-gulono-*γ*-lactone in the VC synthesis pathway. Wild-type mice have ability to synthesise VC in their bodies through the SMP30. Accordingly, SMP30-KO mice demonstrate an onset of scurvy when given a VC-deficient diet^(28)^ similar to humans; they are thus a useful subject for studying the role of VC *in vivo*. However, the interaction between VC and VE in each tissue of mice has not been examined, and their tissue specificity remains unclear, even though some functional actions of VC and VE have been examined in mice^(29,30)^.

Therefore, this study aimed to surrogate investigate the interaction between VC and VE *in vivo* by analyses of their concentrations, the gene expression of associated transporters and GSH level in various tissues of SMP30-KO mice.

## Materials and methods

### Animal experiments

All experimental procedures using laboratory animals were approved by the Animal Care and Use Committee of Tokyo Metropolitan Institute of Gerontology (TMIG) (Approval Number: 18 037) and were in accordance with the guidelines for the Care and Use of Laboratory Animals of TMIG. Sample size calculation of this animal study was determined by G power software^(31)^. SMP30-KO mice were generated using a gene targeting technique as described previously^(32)^. Female SMP30^−/−^ mice were mated with male SMP30^Y/−^ mice to produce SMP30-KO mice. Male SMP30-KO mice were used in this study. The animals were housed under the conditions of 4 mice per cage, at 22 ± 1°C and 55 ± 5 % humidity under a 12 h light/dark cycle in the specific pathogen free environment. After weaning at 4 weeks of age, all mice were provided with water containing 1·5 g/l VC (DSM Japan, Tokyo, Japan) and fed a CRF-1 diet (Oriental Yeast Co., Ltd, Tokyo, Japan), which contained 120 mg/kg VC and 203 mg/kg VE as the normal diet for a week. At 5 weeks of age, the mice were divided into the following four groups with equal average weight: VC+/VE+, VC+/VE–, VC–/VE+ and VC–/VE– (each group, *n* 8). The VC+/VE+ group was fed a diet containing 500 mg/kg VE (Funabashi Farm Co., Ltd, Chiba, Japan) and provided with water containing 1·5 g/l VC. The VC+/VE– group was fed a diet without VE (Funabashi Farm Co., Ltd) and provided with water containing 1·5 g/l VC. The VC–/VE+ group was fed a diet containing 500 mg/kg VE and provided with water without VC. The VC–/VE– group was fed a diet without VE and provided with water without VC. These VE-supplemented or -depleted chows did not contain VC. All drinking water was supplemented with 10 µM EDTA to stabilise the VC; the drinking water and chow were changed every three or four days. The chow and drinking water were provided *ad libitum,* and consumption was measured in each cage. All mice were weighed once a week and were housed for a month. At 9 weeks of age, mice were anaesthetised with isoflurane (Pfizer, Tokyo, Japan) after fasting for 3 h, and blood was collected from the inferior vena cava. The collected blood was gently mixed with EDTA and centrifuged at 800 × g for 15 min at 4°C. The supernatants were collected as plasma for further analysis. Subsequently, mice were systemically perfused with ice-cold PBS through the left ventricle to wash out the blood cells. The liver, heart, lungs, pancreas, kidneys, testes, epididymal fat, cerebrum and cerebellum were collected, weighed, frozen in liquid nitrogen and stored at –80°C until used.

### Blood biochemical examination

Albumin, aspartate aminotransferase, alanine aminotransferase, total cholesterol, TAG, NEFA, LDL-cholesterol and HDL-cholesterol in plasma were assessed at Nagahama Life Science Laboratory (Oriental Yeast Co., Ltd).

### Vitamin E measurement

Tissues were homogenised in PBS using a teflon pestle homogeniser. The homogenates, plasma and *α*-tocopherol standard (Tama Biochemical Co., Ltd., Tokyo, Japan) were mixed with 70 % ethanol containing 6·9 g/l 2,6-Di-tert-butyl-p-cresol (Tokyo Chemical Industry Co., Ltd., Tokyo, Japan) and *dl*-tocol (Tama Biochemical Co., Ltd.) as an internal standard. After adding hexane, the mixtures were rotated for 30 min at room temperature and subsequently centrifuged at 20 000 × g for 5 min. The upper hexane layer containing VE and *dl*-tocol was collected. The hexane was vaporised by N_2_ spraying on a heat block at 40°C, and the residue was dissolved in ethanol. Both VE and *dl*-tocol contents were determined using an HPLC (Waters 2695 separation module, Nihon Waters, Tokyo, Japan) equipped with a Cadenza CD-C18 4·6 mm ID × 150 mm (particle 3 µm, Imtakt Corporation, Kyoto, Japan) column combined with a Cadenza CD-C18 2 mm ID × 5 mm column (particle 3 µm, Imtakt Corporation). The mobile phase was 90 % ethanol containing 50 mM NaClO_4_ and flowed at a rate of 700 µl/min. Electrical signals were recorded using an electrochemical detector (ECD) (Waters 2465 Electrochemical Detector, Nihon Waters). Temperatures for the autosampler, column and ECD were set at 4°C, 40°C and 30°C, respectively. VE levels were normalised to *dl*–tocol and were determined using the VE standard curve.

### Vitamin C measurement

Tissues were homogenised in 5·4 % metaphosphoric acid (MPA, FUJIFILM Wako Pure Chemical Co.) and 1 mM EDTA using a teflon pestle homogeniser. Supernatants collected after centrifugation at 20 000 × g for 15 min at 4°C were moderately diluted with 5 % MPA/EDTA, and samples were treated with 35 mM tris (2-carboxyethyl) phosphine hydrochloride to convert the dehydroascorbic acid (DHA), an oxidised form of VC, to ascorbate. The principle of conversion by tris (2-carboxyethyl) phosphine hydrochloride has been described previously^(33)^. After incubation for 2 h at 4°C, 5 % MPA/EDTA was added to the samples. Samples without tris (2-carboxyethyl) phosphine hydrochloride treatment were also prepared to measure ascorbate alone. Filtrated samples were analysed using an HPLC (Waters 2695 separation module, Nihon Waters) equipped with an Atlantis dC18 5 µm column 4·6 × 150 mm (Nihon Waters) combined with an Atlantis dC18 5 µm guard column 4·6 × 20 mm (Nihon Waters). The mobile phase comprised 50 mM phosphate buffer (pH 2·8), 540 μM EDTA-Na and 2 % methanol, with a flow rate of 1·3 ml/min. Electrical signals were recorded using an ECD (Waters 2465 Electrochemical Detector). Temperatures for the autosampler, column and ECD were set at 4°C, 30°C and 30°C, respectively. Ascorbate levels were determined using an ascorbate (FUJIFILM Wako Pure Chemical Co.) standard curve. DHA content was calculated by subtracting the amounts of ascorbate from the total ascorbate converted by tris (2-carboxyethyl) phosphine hydrochloride. In the present study, the VC level was considered as total ascorbate, including DHA.

### RNA extraction and cDNA synthesis

The liver, heart and kidneys were homogenised in ISOGEN (FUJIFILM Wako Pure Chemical Co.) using a teflon pestle homogeniser, and total RNA was extracted according to the manufacturer’s instructions. RNA concentrations were determined by measuring the absorbance at 260 nm and were confirmed as free from protein contamination by measuring the ratio of absorbance at 260 and 280 nm. cDNA was synthesised from RNA using SuperScript III reverse transcriptase (Invitrogen, Carlsbad) according to the manufacturer’s instructions. The cDNA samples were stored at −80°C until used.

### Polymerase chain reaction

Real-time PCR was performed using the StepOne Plus (Applied Biosystems) and THUNDERBIRD^®^ qPCR Mix (Toyobo), according to the manufacturer’s instructions. Primers targeting *α*TTP (*Ttpa*), ABCA1 (*Abca1*), sodium-dependent vitamin C transporter 1 (SVCT1) (*Slc23a1*), sodium-dependent vitamin C transporter 2 (SVCT2) (*Slc23a2*) and glyceraldehyde-3-phosphate dehydrogenase (*Gapdh*) were purchased from Eurofins Genomics Japan (Tokyo, Japan). The primer sequences are shown in Supplementary Table S1. The amplification protocol consisted of denaturation at 95°C for 1 min, followed by 40 cycles of 95°C for 15 s and 60°C for 1 min. A standard curve was designed for the quantitative analysis of each mRNA expression level; an aliquot of each experimental sample was used to generate the standard curve. Gene expression levels were normalised to those of *Gapdh*.

### Glutathione measurement

The liver, heart and kidneys were homogenised in 5·6 % MPA/EDTA using a teflon pestle homogeniser. Supernatants collected after centrifugation at 21 000 × g for 15 min at 4°C were moderately diluted with 5 % MPA/EDTA. Filtrated samples were analysed using an HPLC (Waters 2695 separation module, Nihon Waters) equipped with a SunFire TM C18 5 µm 4·6 × 150 mm column (Nihon Waters). The mobile phase comprised 100 mM sodium perchlorate monohydrate, 2 % acetonitrile and 0·05 % trifluoroacetate monohydrate, with a flow rate of 0·7 ml/min. Electrical signals were recorded using an ECD (Waters 2465 Electrochemical Detector). Temperatures for the autosampler, column and ECD were at 4°C, 30°C and 30°C, respectively. GSH levels were determined using an L-glutathione reduced (Sigma-Aldrich) standard curve.

### Statistical analysis

Data are expressed as the mean ± standard error of the mean (sem). Significant differences between each group were analysed by ANOVA followed by Tukey’s test using GraphPad Prism 6 (GraphPad Software Inc.). Two-way ANOVA was also performed using GraphPad Prism 6 to analyse the interaction between VC and VE. Statistical differences were considered significant at *P* < 0·05.

## Results

### Body and tissue weights

During the experimental period, no significant differences were observed in the consumption of drinking water and chow among the four groups (online Supplemental Fig. S1). The mean body and tissue weights are shown in Table 1. Body weights were not significantly different among the four groups. The liver weights of the VC–/VE+ and VC–/VE– groups were higher than those of the VC+/VE+ and VC+/VE– groups. The kidneys weight of the VC+/VE– group was higher than that of the VC+/VE+ group. In the heart, lungs, pancreas, testes, epididymal fat, cerebrum and cerebellum, no significant differences were observed among the four groups.

**Table 1.** Body and tissue weights

Group	VC+/VE+		VC+/VE–		VC–/VE+		VC–/VE–	
	Mean	sem	Mean	sem	Mean	sem	Mean	sem
Initial body weight (g)	21·2	0·4	21·2	0·3	21·3	0·5	21·2	0·8
Final body weight (g)	24·2	0·3	25·0	0·4	24·6	0·3	25·4	0·7
Tissue weight (mg/g body weight)								
Liver	40·8	1·2 ^a^	43·3	1·5 ^a^	49·3	1·3 ^b^	49·4	1·5 ^b^
Heart	4·6	0·1	4·8	0·2	4·7	0·1	4·6	0·2
Lungs	6·8	1·0	6·9	0·6	6·5	0·4	7·4	0·7
Pancreas	9·4	0·7	9·9	0·7	11·3	0·7	11·3	0·4
Kidneys	12·4	0·3 ^a^	13·7	0·4 ^b^	13·1	0·2 ^ab^	13·3	0·3 ^ab^
Testes	7·7	0·3	7·7	0·2	7·8	0·2	7·3	0·3
Epididymal fat	24·4	1·9	22·2	2·1	19·5	2·1	20·9	0·8
Cerebrum	12·9	0·2	12·1	0·3	12·6	0·3	12·4	0·5
Cerebellum	2·8	0·1	2·3	0·3	2·5	0·2	2·9	0·4

Data are expressed as mean ± sem (*n* 6–7). Different letters indicate significant differences (*P* < 0·05) among groups by Tukey’s test followed one-way ANOVA.

### Blood biochemical examination

The plasma levels of albumin, aspartate aminotransferase, total cholesterol, TAG, NEFA, LDL-cholesterol and HDL-cholesterol are shown in Table 2. There were no significant differences among the four groups in any of the parameters. Five mice of the total 32 mice that showed abnormal values in the blood test results were excluded from analyses of all experiments.

**Table 2.** Blood biochemical examination

Group	VC+/VE+		VC+/VE–		VC–/VE+		VC–/VE–	
	Mean	sem	Mean	sem	Mean	sem	Mean	sem
ALB (g/dl)	3·1	0	3·0	0	3·1	0	3·2	0
AST (IU/l)	52·3	4·2	46·6	2·7	52·3	5·2	51·3	4·6
ALT (IU/l)	31·1	3·4	21·7	2·0	36·6	5·8	25·7	3·2
T-CHO (mg/dl)	117·7	3·9	102·3	2·1	92·3	1·8	99·3	1·7
TAG (mg/dl)	68·3	2·6	54·3	2·9	77·4	3·1	61·7	4·8
NEFA (μEq/l)	554·3	6·9	490·9	12·5	545·1	14·2	519·0	12·2
LDL-cholesterol (mg/dl)	7·7	0·4	6·6	0·3	5·1	0·2	5·7	0·1
HDL-cholesterol (mg/dl)	58·6	1·4	54·0	0·9	52·9	0·9	55·0	0·9

ALB, albumin; AST, aspartate aminotransferase; ALT, alanine aminotransferase; T-CHO, total cholesterol. Data are expressed as mean ± sem (*n* 6–7). The significant differences between each group were analysed by one-way ANOVA.

### Vitamin E levels in various tissues

**Fig. 1.**
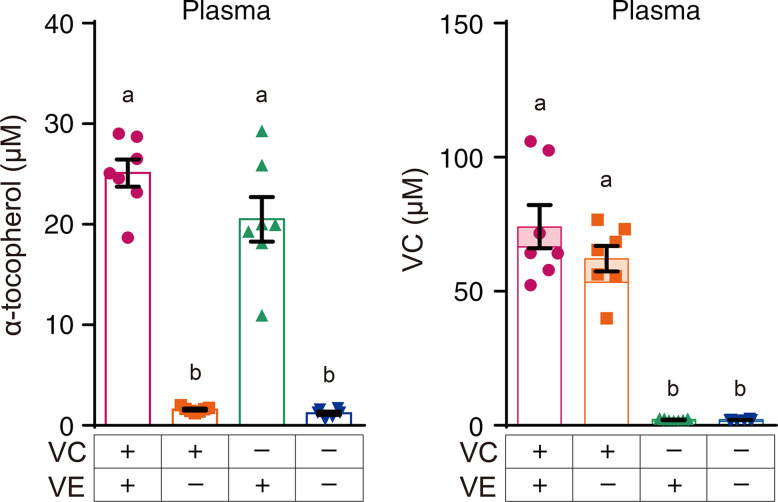
VE and VC concentrations in the plasma of SMP30-KO mice fed with control (VC+/VE+), VE-depleted (VC+/VE–), VC-depleted (VC–/VE+) or simultaneously VE and VC-depleted (VC–/VE–) diets for a month. The painted upper part of VC in each column indicates the DHA level. Data are expressed as the mean ± sem (*n* 6–7). Different letters indicate significant differences (*P* < 0·05) among groups by Tukey’s test following one-way ANOVA.

**Fig. 2.**
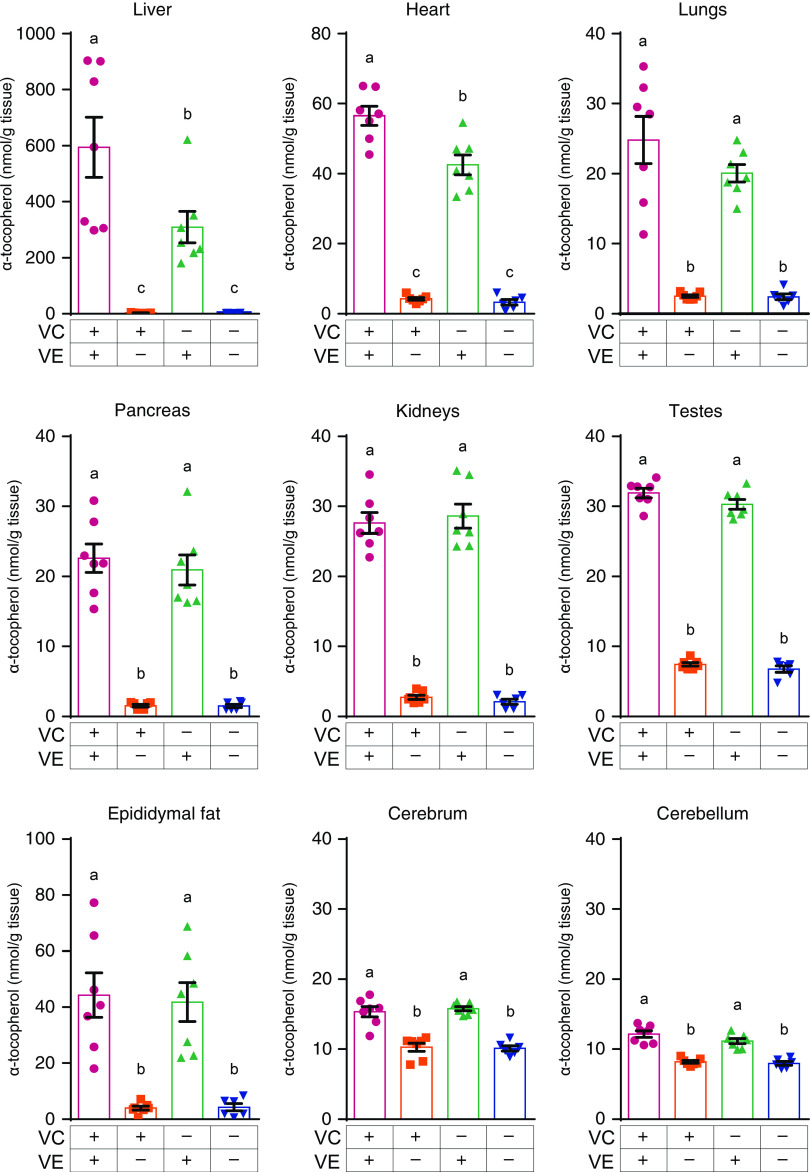
VE concentrations in the liver, heart, lungs, pancreas, kidneys, testes, epididymal fat, cerebrum and cerebellum of SMP30-KO mice fed with control (VC+/VE+), VE-depleted (VC+/VE–), VC-depleted (VC–/VE+) or simultaneously VE and VC-depleted (VC–/VE–) diets for a month. Data are expressed as the mean ± sem (*n* 6–7). Different letters indicate significant differences (*P* < 0·05) among groups by Tukey’s test following one-way ANOVA.

The plasma VE levels of the VC+/VE– and VC–/VE– groups were significantly lower than those of the VC+/VE+ and VC–/VE+ groups, whereas no significant differences were observed between the VC+/VE+ and VC–/VE+ groups (Fig. 1). The VE levels in various tissues are shown in Fig. 2. In all tissues, the VE levels in the VC+/VE– and VC–/VE– groups were lower than those in VC+/VE+ and VC–/VE+ groups. Notably, VE levels in the liver and heart of the VC–/VE+ group were significantly lower than those of the VC+/VE+ group; there were no significant differences in the lungs, pancreas, kidneys, testes, epididymal fat, cerebrum and cerebellum among the groups.

### Vitamin C levels in various tissues

**Fig. 3.**
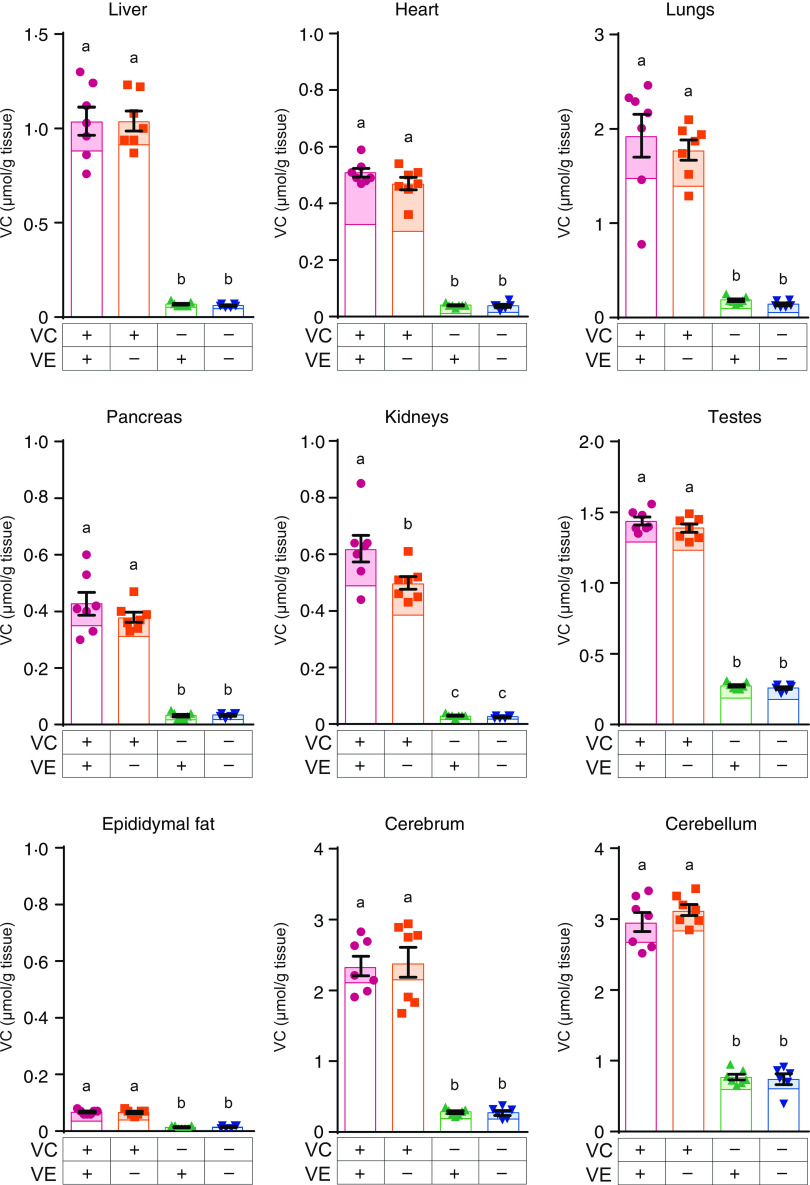
VC concentrations in the liver, heart, lungs, pancreas, kidneys, testes, epididymal fat, cerebrum and cerebellum of SMP30-KO mice fed with control (VC+/VE+), VE-depleted (VC+/VE–), VC-depleted (VC–/VE+) or simultaneously VE and VC-depleted (VC–/VE–) diets for a month. The painted upper part in each column indicates the DHA level. Data are expressed as the mean ± sem (*n* 6–7). Different letters indicate significant differences (*P* < 0·05) among groups by Tukey’s test following one-way ANOVA.

VC levels in the plasma of VC–/VE+ and VC–/VE– groups were significantly lower than those in the VC+/VE+ and VC+/VE– groups (Fig. 1), whereas no significant differences were observed between the VC+/VE+ and VC+/VE– groups. The VC levels in various tissues are shown in Fig. 3. In all tissues, VC levels in the VC–/VE+ and VC–/VE– groups were lower than those in the VC+/VE+ and VC+/VE– groups. Notably, VC levels in the kidneys were significantly lower in the VC+/VE– group than those in the VC+/VE+ group; there were no significant differences in the liver, heart, lungs, pancreas, testes, epididymal fat, cerebrum and cerebellum among the groups.

### Gene expression of transporters associated with vitamin C and vitamin E in the liver, heart and kidneys

**Fig. 4.**
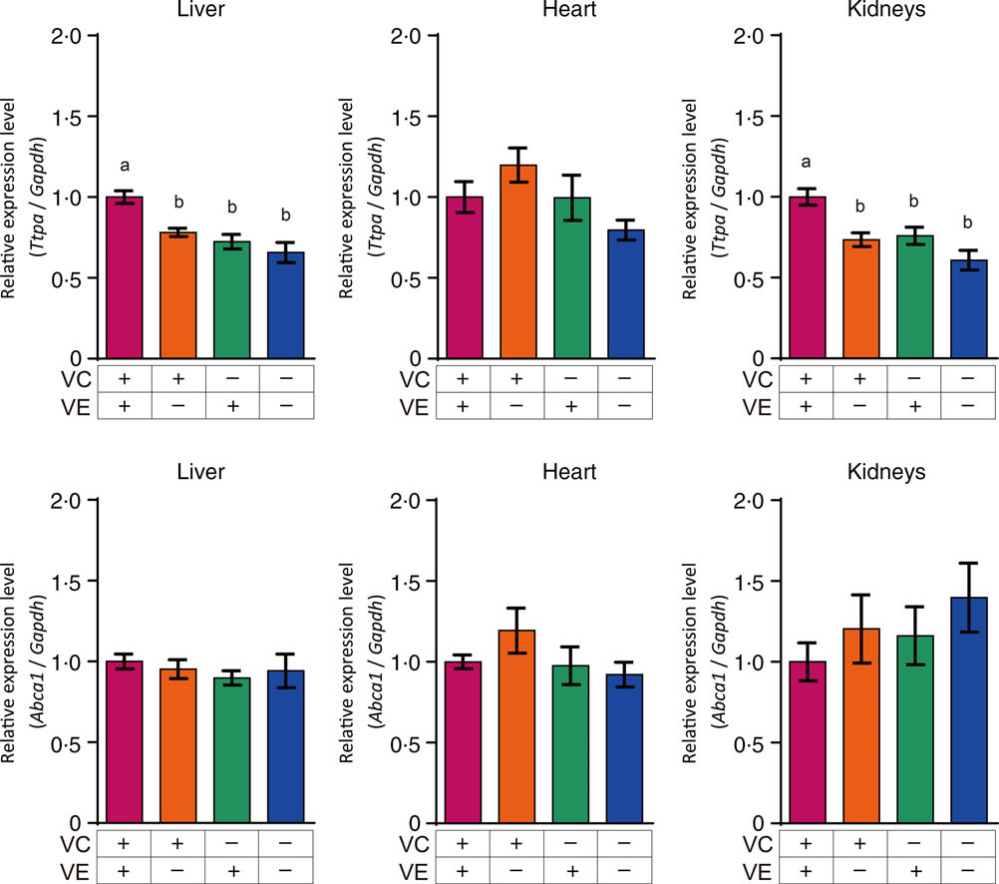
Relative expression levels of *α*TTP and ABCA1 in the liver, heart and kidneys of SMP30-KO mice fed with control (VC+/VE+), VE-depleted (VC+/VE–), VC-depleted (VC–/VE+) or simultaneously VE and VC-depleted (VC–/VE–) diets for a month. Data are expressed as the mean ± sem (*n* 6–7). Different letters indicate significant differences (*P* < 0·05) among groups by Tukey’s test following one-way ANOVA.

**Fig. 5.**
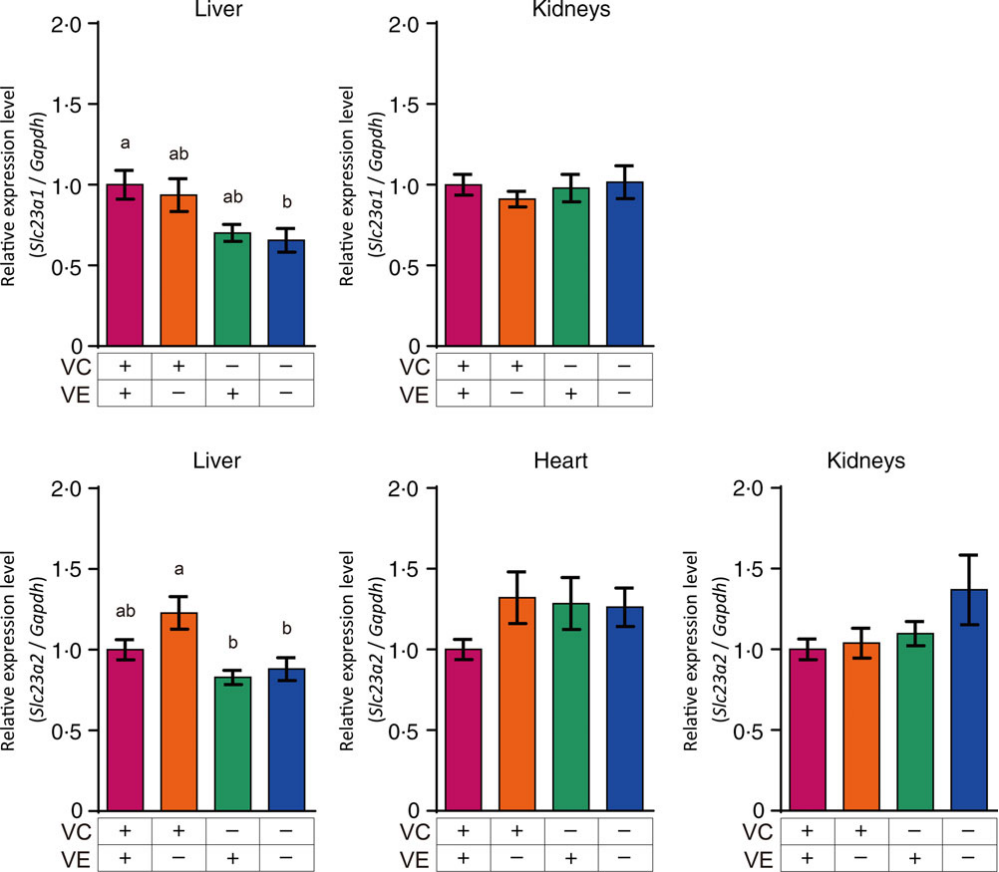
Relative expression levels of SVCT1 and SVCT2 in the liver, heart and kidneys of SMP30-KO mice fed with control (VC+/VE+), VE-depleted (VC+/VE–), VC-depleted (VC–/VE+) or simultaneously VE and VC-depleted (VC–/VE–) diets for a month. Data are expressed as the mean ± sem (*n* 6–7). Different letters indicate significant differences (*P* < 0·05) among groups by Tukey’s test following one-way ANOVA.

The relative expression levels of *α*TTP (*Ttpa*) and ABCA1 (*Abca1*) in the liver, heart and kidneys are shown in Fig. 4. The *α*TTP expression levels in the liver and kidneys of the VC+/VE–, VC–/VE+ and VC–/VE– groups were lower than those in the VC+/VE+ group, and no significant difference was observed in the *α*TTP expression level in the heart. Furthermore, there were no significant differences in the ABCA1 expression levels in the liver, heart and kidneys among the four groups. The relative expression levels of SVCT1 (*Slc23a1*) and SVCT2 (*Slc23a2*) in the liver, heart and kidneys are shown in Fig. 5. SVCT1 expression levels in the liver were lower in the VC–/VE– group than those in the VC+/VE+ group, and there were no significant differences in SVCT1 expression levels in the kidneys. Additionally, SVCT1 expression was not detected in the heart. SVCT2 expression levels in the liver of the VC–/VE+ and VC–/VE– groups were lower than those in the VC+/VE– group; there were no significant differences in the SVCT2 expression levels in the heart and kidneys among the groups.

### Glutathione levels in the liver, heart and kidneys

**Fig. 6.**
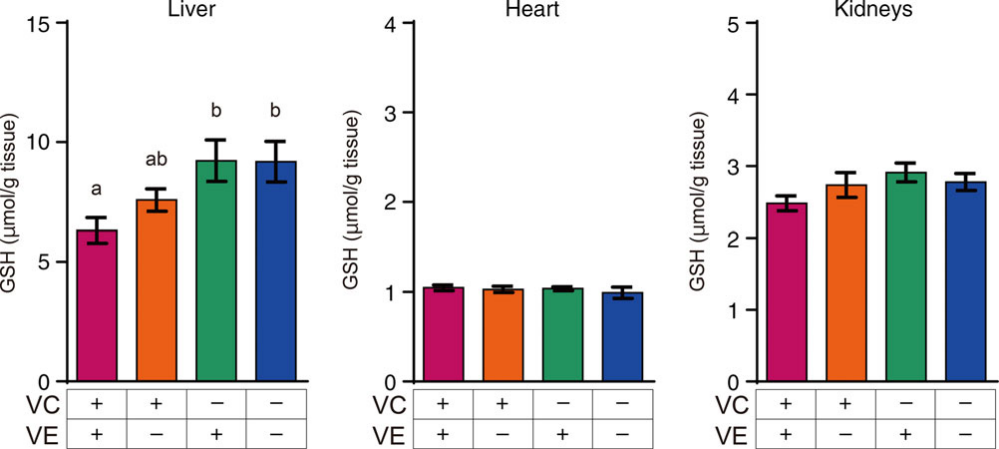
GSH levels in the liver, heart and kidneys of SMP30-KO mice fed with control (VC+/VE+), VE-depleted (VC+/VE–), VC-depleted (VC–/VE+) or simultaneously VE and VC-depleted (VC–/VE–) diets for a month. Data are expressed as the mean ± sem (*n* 6–7). Different letters indicate significant differences (*P* < 0·05) among groups by Tukey’s test following one-way ANOVA.

The GSH levels in the liver, heart and kidneys are shown in Fig. 6. GSH levels in the liver of VC–/VE+ and VC–/VE– groups were significantly higher than those in the VC+/VE+ group. On the other hand, there were no significant differences in the GSH levels in the heart and kidneys.

## Discussion

In this study, the interaction between VC and VE with respect to their concentrations, the gene expression of their transporters and GSH level in various tissues were examined using SMP30-KO mice. Based on the intake of mice (online Supplementary Fig. S1), the converted doses of VC and VE in a human (60 kg body weight) were estimated approximately 1000 mg/d VC and 300 mg/d VE according to the FDA guidance. These doses are clinically taken as supplements. SMP30-KO mice are deficient in VC synthesis and develop scurvy along with a decrease in body weight after 2 months on a VC-depleted diet^(28)^. Combined deficiency of VC and VE is reported to cause paralysis of the limbs and early death in guinea pigs^(34)^. In the present study, we aimed to investigate the interaction between VC and VE in mice before they developed serious scurvy. Comparison of blood biochemical parameters among the groups revealed no significant differences. Furthermore, there were no differences in the mean body weights among the groups. Notably, liver weights in the VC–/VE+ and VC–/VE– groups were higher than those in VC+/VE+ and VC+/VE– groups. This suggests that VC deficiency in SMP30-KO mice affects their liver weights. Ikeda *et al.*^(35)^ revealed that VC deficiency in osteogenic disorder Shionogi rats altered the gene expression of acute-phase proteins in the liver. Moreover, our previous study using transcriptome microarray analyses indicated that VC deficiency increases redox-related and lipid metabolism-related factors including a late-limiting enzyme of the bile acid biosynthesis pathway in the liver of SMP30-KO mice^(36)^. These findings may be related to the results of this study.

Interaction between VC and VE was not clearly observed in the plasma. This result is inconsistent with previous studies in rats^(18)^ and humans^(22)^ and could be due to differences in the dose, period and biological species. Remarkably, low VE levels were observed in the liver and heart in the VC–/VE+ group compared with those in the VC+/VE+ group. Additionally, statistical interaction of VC and VE was observed in the liver and heart, whereas there were no significant differences in the other tissues tested. Dietary VE is absorbed in the small intestine and is secreted with triacylglycerol-rich chylomicrons into the lymph and blood, followed by transport to the liver. VE is incorporated into very-LDL by ABCA1 and is transported to peripheral tissues by lipoprotein through *α*TTP^(37)^. Mutations in the gene coding for *α*TTP cause ataxia with isolated vitamin E deficiency^(38)^. *α*TTP is expressed mainly in the liver, indicating that VE metabolism is largely regulated in the liver. In this study, the expression *Ttpa* gene, which encodes *α*TTP, was decreased by the lack of VC and/or VE in the liver. However, a similar result was not seen in the heart. *α*TTP expression in the kidneys was also affected by VC and VE deficiency, though the VE concentration was not changed by VC deficiency in the kidneys. It is thus thought that VE as well as VC may affect *Ttpa* gene expression in the kidneys. On the contrary, ABCA1 seems to be expressed constitutively to maintain lipid metabolism and is not influenced by VC and VE levels, similar to a previous report in wild-type mice^(39)^.

As points to be considered, the reaction rate between VE radicals and VC may be influenced by pH^(40)^, and the reaction may not occur under anaerobic conditions^(41)^. Moreover, hydrogen, but not VC, increases the regeneration of VE in rat adipose tissues, but a similar result was not observed in the liver^(42)^. These reports suggest that the VE regeneration mechanism differs by the tissue and condition. Furthermore, there is a possibility that studies in different animal models may provide different results although we have not tested in other models.

A significant difference in VC concentrations between the VC+/VE– and VC+/VE+ groups was observed only in the kidneys. DHA, an oxidised form of VC, was not markedly different. In the present study, DHA levels were measured for the first time in the investigation of interaction between VC and VE *in vivo*. DHA is considered equivalent to VC because intracellular DHA is rapidly reduced to VC^(43,44)^. Therefore, we regarded VC as including DHA in addition to ascorbate. Our results suggest that VE has a sparing effect on VC in the kidneys, but this interaction was not observed in the other tissues tested. The possibility that VE spares VC has been described previously in the plasma and some tissues from other species^(18,22)^; however, this study showed the specificity of this interaction in the kidneys in mice. In this study, the gene expression levels of VC transporters, SVCT1 and SVCT2, were also analysed. Although the pharmacokinetics of VC are complex, the majority of tissue distribution and renal reuptake are handled by the SVCTs^(45)^. In the liver, it is suggested that SVCT1 expression is affected by VC and VE deficiency, and that SVCT2 expression is affected only by VC deficiency. However, the expression of these genes was not changed in the kidneys. A previous study has also shown that SVCT1 and SVCT2 expression in the kidneys is not affected by VC depletion in SMP30-KO mice; however, these were increased in the liver of SMP30-KO mice upon VC depletion^(46)^. Gene expression of vitamin transporters is thought to be affected by their specific vitamins as well as other factors such as other nutrients, fasting time and aging^(39,47)^. Therefore, one of the limitations of this study is that multiple time points were not examined. Moreover, it is thought that other factors such as renal functions, metabolites and oxidative stress might be involved in the sparing effect on VC by VE in the kidneys while we have not examined.

Interestingly, VC levels in the liver and heart were not decreased by VE coexistence despite the sparing effect of VC on VE. Additionally, GSH levels were significantly high in VC–/VE+ and VC–/VE– groups in the liver. These results suggest that VC might be regenerated by GSH in the liver in VC+/VE+ group, while there is another possibility that GSH levels were increased to replace VC by VC deficient^(48)^. DHA is recycled by GSH and nicotinamide-adenine dinucleotide phosphate^(49)^, suggesting that VC is available for VE recycling, but there are some experimental reports on VE regeneration by other antioxidants^(50)^. Moreover, the VC level in the heart was found to be low, but VC spared VE, suggesting that VE might be required or that oxidative stress might be high in the heart. Further studies including detailed mechanisms are thus required. In addition, highly conserved VE and VC levels were observed in the cerebrum and cerebellum, respectively, despite their depletion for a month. These observations could emphasise their important role in neuroprotection^(51,52)^.

Since the suggestion of interaction between VC and VE, several clinical studies on combined vitamins have been performed. Supplementation of VC and VE has been shown to be useful in preventing bone loss linked to oxidative stress in the elderly^(53)^. A recent meta-analysis also indicated beneficial effects of VC and VE co-supplementation on serum C-reactive protein, an important biomarker for predicting diseases, in participants ≥ 30 years of age but not in young subjects^(54)^. Further, a randomised clinical trial showed that co-administration of VC and VE may improve arterial blood gas parameters and reduce intensive care unit stay in patients with lung contusion^(55)^. Thus, co-supplementation of VC and VE has been expected to improve several diseases by decreasing oxidative stress^(56,57)^, and our results support these mechanisms kinetically.

In conclusion, our study provides a new finding that VC spares VE mainly in the liver and heart and that VE spares VC in the kidneys of SMP30-KO mice via different mechanisms. Further development based on the interactive effects of VC and VE is desired to design a nutritional approach to diseases.

## Supplementary material

For supplementary material accompanying this paper visit https://doi.org/10.1017/S0007114521004384. click here to view supplementary material
