# Supplementary material for: Determination of tissue-specific interaction between vitamin C and vitamin E in vivo using senescence marker protein-30 knockout mice as a vitamin C synthesis deficiency model
Source: Br J Nutr. 2021 Nov 2;128(6):993–1003. doi: 10.1017/S0007114521004384 (PMC9381305; doi:10.1017/S0007114521004384)
Supplement: Supplementary file 1 [file S0007114521004384sup.zip › S0007114521004384sup002.docx]

Table S1. Primer sequences

| Gene Name | Sequence of forward primer (5' - 3') | Sequence of reverse primer (5' - 3') |
| --- | --- | --- |
| *Abca1* | CCATACCGAAACTCGTTCACC | CCGCAGACATCCTTCAGAATC |
| *Ttpa* | ACTCTTTACCGCCATATTCCC | TTTCCATGCTGTCTTCTCCA |
| *Slc23a1* | CAGCAGGGACTTCCACCA | CCACACAGGTGAAGATGGTA |
| *Slc23a2* | AACGGCAGAGCTGTTGGA | GAAAATCGTCAGCATGGCAA |
| *Gapdh* | TGAAGCAGGCATCTGAGGG | CGAAGGTGGAAGAGTGGGAG |

ABCA1 (*Abca1*), αTTP (*Ttpa*), SVCT1 (*Slc23a1*), SVCT2 (*Slc23a2*), and glyceraldehyde-3-phosphate dehydrogenase (*Gapdh*).
