# Supplementary figures and images for: Determination of tissue-specific interaction between vitamin C and vitamin E in vivo using senescence marker protein-30 knockout mice as a vitamin C synthesis deficiency model
Source: Br J Nutr. 2021 Nov 2;128(6):993–1003. doi: 10.1017/S0007114521004384 (PMC9381305; doi:10.1017/S0007114521004384)

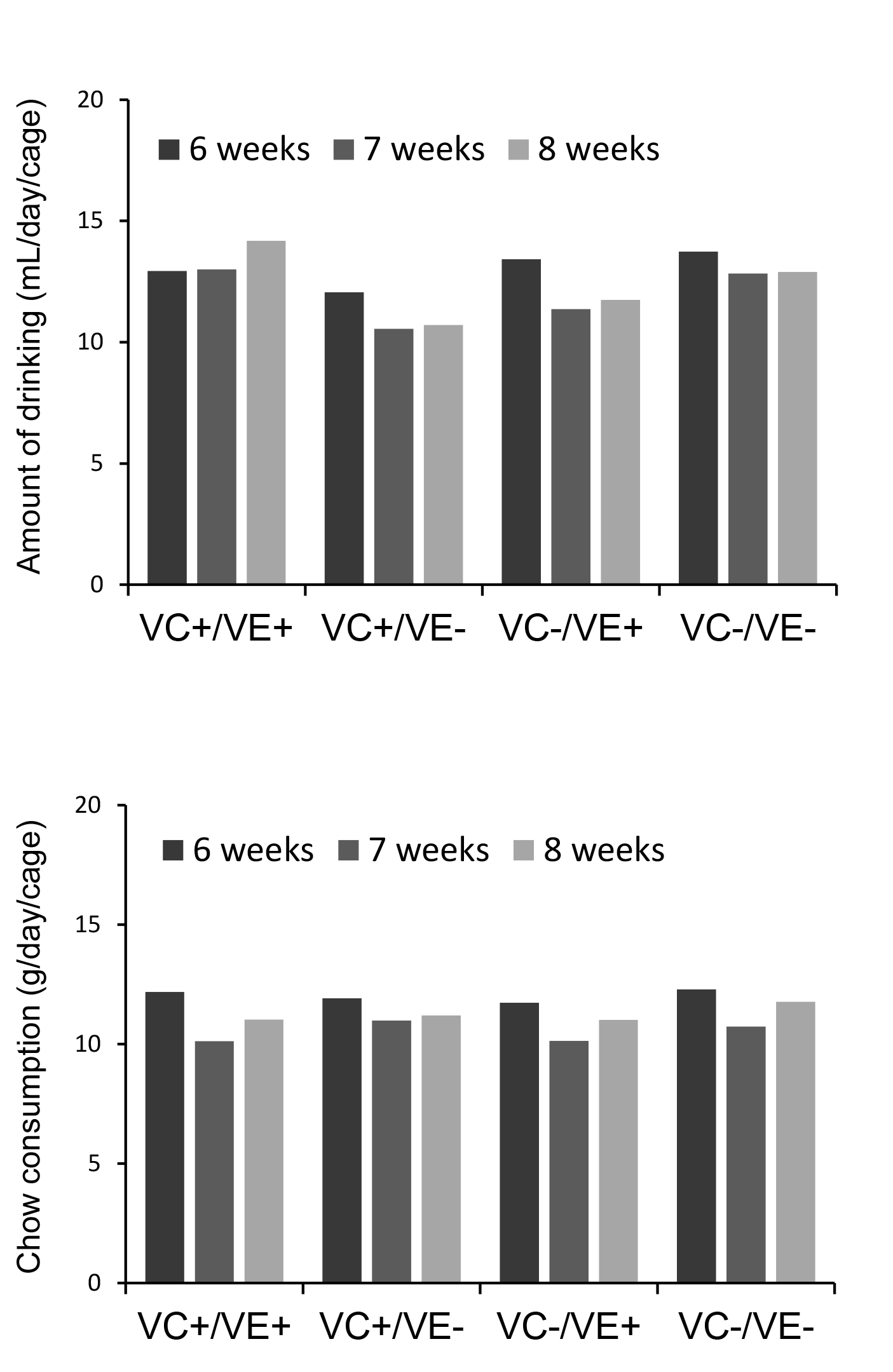

Supplement: Supplementary file 1 [file S0007114521004384sup.zip › S0007114521004384sup001.tif]
